# Improving Estimation of Layer Thickness and Identification of Slicer for 3D Printing Forensics

**DOI:** 10.3390/s23198250

**Published:** 2023-10-05

**Authors:** Bo Seok Shim, Jong-Uk Hou

**Affiliations:** Division of Software, Hallym University, Chuncheon 24252, Republic of Korea; bycicle55@naver.com

**Keywords:** 3D printing, image forensics, artificial intelligence

## Abstract

This study emphasizes the significance of estimating the layer thickness and identifying slicer programs in the realm of 3D printing forensics. With the progress in 3D printing technology, precise estimation of the layer thickness has become crucial. However, previous research on layer thickness estimation has mainly treated the problem as a classification task, which is inadequate for continuous layer thickness parameters. Furthermore, previous studies have concentrated on hardware-based printer identification, but the identification of slicer programs through 3D objects is a vital aspect of the software domain and can provide valuable clues for 3D printing forensics. In this study, a regression-based approach utilizing a vision transformer model was proposed. Experiments conducted on the SI3DP++ dataset demonstrated that the proposed model could handle a broad range of data and outperform the current classification models. Additionally, this study proposed a new research direction by introducing slicer program identification, which significantly contributes to the field of 3D printing forensics.

## 1. Introduction

Additive Manufacturing (AM), commonly known as 3D printing, is a technology that fabricates 3D objects by building them layer by layer using digital 3D modeling files [1]. This innovative technology enables the manufacturing of products with intricate designs that cater to individual preferences, which is challenging to accomplish using traditional methods. AM offers numerous advantages including rapid prototyping, reduced cost of production, and improved accessibility. Consequently, it has revolutionized production and product design in various industries, such as aerospace, automotive, medical, and manufacturing [2]. The 3D printing market has been growing consistently, with global efforts made to implement the 3D printing technology in various industries and everyday life. Notably, in the United States, major manufacturing companies have launched the AM Forward program, a collaborative initiative aimed at promoting the growth of AM within the country [3]. In particular, this program supports small- and medium-sized manufacturers involved in layered manufacturing using 3D printing. Significant advancements in technology and the widespread adoption of consumer-grade 3D printers have made them more accessible to individuals and organizations. In addition, Ai Build developed the Talk to AISync artificial intelligence program, which combines the capabilities of the large-scale language model ChatGPT with 3D printing software [4]. The goal is to reduce the barriers to entry into the 3D printing field and optimize the production of complex-shaped products at a rapid pace, thus further contributing to its advancement. As 3D printing technology continues to progress, 3D printers are expected to become more user-friendly and to play a crucial role in the interaction between businesses and individuals, where they can offer a flexible and efficient means of producing customized products and components.

However, the significant advantages of the 3D printing technology in various industries are counterbalanced by crucial security and ethical gaps that require attention. Currently, established guidelines and regulations for 3D printing technology are lacking, leading to issues such as intellectual property infringement, counterfeiting, illegal replication, and cybersecurity concerns [5]. Instances of abuse of 3D printing can be categorized into two primary areas: digital and physical. In the 3D printing process, the digital domain involves the use of computer-aided design (CAD) software to create digital files that are subsequently transferred between computers and 3D printers. This digital domain encompasses various aspects of digital information security, such as authentication and encryption. In the realm of digital crime, there are ongoing legal cases where individuals within online communities that share designs, such as Thingiverse and Cubify, are suspected of illegally utilizing digital files for personal gain [6]. In addition, in some instances, 3D printers are used to create fake fingerprints and face masks, which are then used to hack mobile phones and security systems [7]. Furthermore, 3D printers have been employed to manufacture ATM card skimming devices, leading to ATM fraud [7].

The physical domain represents the tangible characteristics of 3D printers and printed 3D objects; it encompasses the materials used and the resulting outputs. A prominent example of abuse in the physical domain is an illegal 3D printed firearm, commonly referred to as a “ghost gun” [8]. The 3D printing technology enables the covert manufacturing of such firearms. Moreover, these firearms are unregistered and can be challenging to detect using metal detectors, making them difficult to trace. Instances of loss of the Transportation Security Administration (TSA) master key has created concerns regarding the potential manufacturing of 3D printed keys that could compromise the security of TSA locks [7]. Recently, there has been a surge in crimes involving individuals downloading vehicle license plate files from the Internet and using 3D printers to produce counterfeit license plates, enabling them to evade tracking [9]. This form of crime poses a serious threat to legitimate vehicle owners, as they may experience significant harm and may be burdened with proving their innocence. Furthermore, concerns have been raised regarding the proliferation of pirated content that infringes upon intellectual property rights as well as the potential risks associated with the illegal or unethical application of medical procedures.

Furthermore, as criminals have gained the ability to create sophisticated objects using consumer-level printers, these problems have become more widespread and difficult to address [10]. Therefore, the precise analysis of physical evidence in the field is significant in identifying the source and production processes of 3D objects. In addition, 3D replicas prepared by scanning the sites of injury of victims have been considered crucial evidence for perpetrator identification in criminal trials [5]. Research on 3D printing forensics is necessary to ensure legal accountability for the actions of organizations and individuals. Consequently, forensic research has been undertaken to address the above concerns in both the digital and physical domains.

Forensic research based on source identification is crucial for determining the authenticity of an incident. Over the years, research has been conducted on various objects including printers, cameras, digital files, and UAVs [11,12,13]. In recent years, as the 3D printing market has grown and diversified, source identification in 3D printing has emerged as a significant requirement [14]. Three-dimansional printing forensics investigates and analyzes printed materials to verify their origin and authenticity and to identify the entities involved. This type of research can be broadly categorized into two main areas. The first area involves tracing specific printers by leveraging the unique patterns or fingerprints of individual printers, which are based on subtle differences in the print quality [15,16,17]. The second area involves incorporating markers such as QR codes, watermarks, or tags into 3D digital files or objects during printing to identify the printing device used [18,19,20].

**Figure 1:**
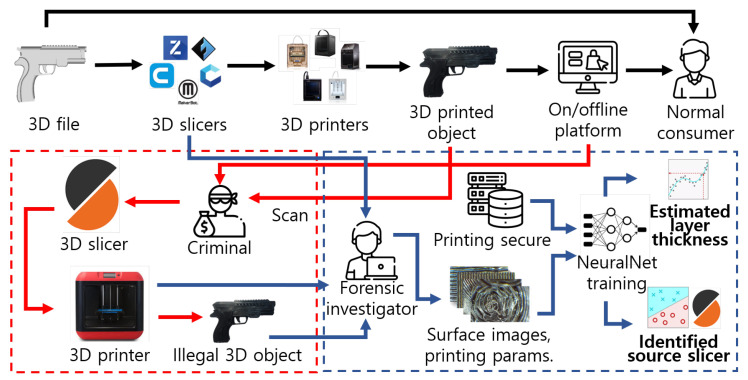
Forensic process of investigating illegally produced manufactured 3D products by criminals through various pathways.

Two main approaches to FDM-based 3D printer identification share similarities with that of our study. In the context of watermarking techniques, Delmotte et al. [20] developed invisible watermarks by subtly modifying the layer thickness. In the medical field, Druelle et al. [18] ensured the distinguishability of printed objects by engraving unique IDs on them. Hou et al. [19] proposed a technique for embedding watermarks by analyzing layer ring artifacts. However, forensic techniques, such as watermarks, have limited applicability owing to specific environmental requirements. Furthermore, the advancement of fusion technologies of chatGPT and 3D printing software [4] has allowed unrestricted manipulation of 3D digital files. Thus, the effectiveness of traditional digital forensic methods such as watermarking is expected to be limited, as depicted in Figure 1.

In contrast, Peng et al. [15] researched the identification of 3D printers by leveraging the texture features of the edges and contours of 3D printed objects. Similarly, Shim et al. [16,21] introduced an open benchmark dataset for 3D printer identification. They extended this identification method beyond printers to include filaments, devices, and layer thicknesses. Kubo et al. [17] identified 3D objects by analyzing variations in auditory periodicity patterns resulting from differences in internal structural patterns. Separate studies were proposed for 3D printer identification based on hot-end temperature comparison [22]. There is a need for research in the physical domain to identify 3D printing sources using traces and patterns of evidence, thereby enabling tracking of criminals. Such research should focus on developing methods for identifying and tracing the origin of 3D printed objects with the objective of providing valuable information for forensic investigations. However, fingerprint-based forensic research approaches commonly rely on classification methods, which can lead to an exponential increase in computational memory when dealing with continuous printing parameters, thereby making accurate predictions challenging. Therefore, further research is required to estimate the continuous variables for precise identification in 3D printing processes.

Previous research on 3D printing forensics has mainly focused on hardware-based printer identification through the analysis of printed objects. This is because the performance and characteristics of a 3D printer directly influence the 3D printed output, making 3D printer identification a crucial aspect of 3D printing forensics. However, identifying slicer programs using 3D objects is a key point for source identification in the software domain and can serve as an important clue in 3D printer identification [23,24]. Moreover, it is worth noting that exploring the characteristics of various slicer programs and researching methods to identify the source equipment from the information obtained from the 3D printed object represent significant focus areas for future research in 3D printing forensics.

In 3D printing, layer thickness has various effects on tensile strength, flexural strength, surface quality, and dimensional accuracy [25]. As the layer thickness decreases, the number of layers increases, leading to higher consistency in the specimen and increased tensile strength. The change in flexural strength can also vary depending on the thickness of each layer, allowing for specific patterns to be displayed. Too small layer thickness can increase printing time and the possibility of surface defects. However, most of the research related to layer thickness in 3D printing so far has focused on studies for better quality output [25,26,27]. Furthermore, layer thickness estimation has been less explored in the past because of the limited sophistication of 3D printers. As 3D printing technology advances, algorithms for precise estimation of layer thickness are becoming essential. However, existing studies [20] mainly treat this approach as a means of object discrimination rather than focusing on the layer thickness estimation itself. The methods adopted in these studies are not suitable for handling the continuous variable nature of the layer thickness.

In this study, we propose a novel approach for estimating the layer thickness, which is a crucial factor in achieving high-quality outputs in 3D printing. Given the variability in the achievable layer thickness across different printers, we propose a detailed parameter detection model based on the vision transformer (ViT) [28] architecture to accurately estimate these parameters. By analyzing the surfaces of printed 3D objects using the SI3DP++ dataset [16] and training the ViT to learn the relationships and patterns between the patches in each image, we improve the performance of the regression tasks. We emphasize the significance of this pioneering research on slicer program identification, which introduces a new perspective in the realm of software-based 3D printing forensics. Furthermore, we validate the robustness of our algorithms from the perspective of 3D printing identification by comparing them with existing research.

The main contributions of our paper are as follows:

1. The paper proposes a new regression-based approach using a vision transformer model for layer thickness estimation in 3D printing forensics, which outperforms previous methods and can handle a broader range of data.
2. The paper introduces slicing program identification as a new research direction in 3D printing forensics, which can provide valuable clues in identifying the source of 3D printed objects.
3. The paper emphasizes the importance of collecting more detailed datasets for the advancement of 3D printing forensics, which can lead to more effective tools and techniques for forensic investigators.

## 2. Background

### 2.1. 3D Printer Categorization

The 3D printing process involves the design of a 3D model and the configuration of various settings, including layer thickness, shell count, printing speed, nozzle diameter, and the use of supports using slicer software. The prepared model is then sent to a 3D printer for fabrication. Three-dimensional printers can be categorized based on criteria such as technology, size, price, and functionality. A commonly used technology is fused deposition modeling (FDM), which uses a thermoplastic filament. The filament is heated by an extruder and then extruded layer by layer onto the print bed using a print head attached to the x-, y-, and z-axis. Other 3D printing technologies include stereolithography (SLA), which uses lasers to solidify liquid resin into desired shapes; selective laser sintering, which employs lasers to fuse powdered materials; digital light processing, which uses projectors instead of lasers to cure liquid resin; and the PolyJet technology, which deposits layers of liquid photopolymer that are instantly cured using UV light [1]. Each of these methods has unique characteristics and applications in 3D printing. Various approaches have been used for 3D printer identification, such as SLA, which differs from the FDM technology used in our research. Researchers have focused on identifying SLA-based 3D printers using parameters such as the printing temperature and equipment distortion [29]. But among these, FDM printers are relatively affordable when compared with other printing technologies, making them a cost-effective option for small businesses and individuals [30]. Additionally, FDM printers can work with a variety of materials, such as PLA, ABS, and nylon, and are user-friendly, making them suitable for beginners.

Three-dimensional printers range from large industrial machines to small desktop models, with prices differing based on the machine size and purpose [31]. Industrial 3D printers, often referred to as large-scale printers, are designed for professional applications and are capable of printing sizable objects but at high costs. These machines are specifically designed for professional use and employed by businesses and organizations to handle complex projects. In contrast, consumer-grade 3D printers are much more affordable and compact, making them suitable for home use, and primarily cater to educators and individuals involved in small-scale projects. Although consumer-level printers may not match the print quality of industrial printers, they can produce high-quality prints with excellent resolution and fine detail. This characteristic makes them easily accessible to a wide range of individuals, providing opportunities for various purposes, including potential misuse, such as the production of counterfeit goods, illegal weapons, and their unauthorized distribution [10]. Furthermore, it is important to note that the existing benchmark datasets for 3D printer identification are currently limited to the FDM printing method, specifically the SI3DP++ dataset [16]. Therefore, in this study, we assume a scenario based on the FDM method and consumer-level printers as our underlying premise.

### 2.2. 3D Printing Characterization Factors

Previous identification studies have established the validity of using the characteristics and attributes of the target subjects as the basis for research, thereby establishing credibility. For camera identification, features such as sensor pattern noise, lens distortion, JPEG quantization, and color filter array have been analyzed [11]. Diverse sensor features in the processing stage, such as microscopy, ink analysis, and dot patterns, have been examined for printer identification [12]. Similarly, in the domain of 3D printing technology, the characterization factors at each stage are broadly categorized into software and hardware domains. In this section, we analyze the characterization factors specific to the 3D printing domain with focus on noise and periodic patterns.

#### 2.2.1. Software Domain

To transform 3D digital files obtained from design-sharing websites or created through CAD programs into physical objects, consumers must undergo a slicing process that converts the files into G-code files using slicer software. The software domain plays a significant role in representing the characterization factors, particularly in the 3D digital files and slicer program [23]. Differences in the personal abilities and expressive skills of individuals can lead to variations in the creation of the same design using CAD programs. This was validated through the identification and analysis of scan reprint data from the SI3DP++ dataset [16], which demonstrated its potential for classifying different 3D digital files. Furthermore, although experts have the ability to manipulate G-code and firmware to directly control the output without relying on slicer programs, it can be challenging for less proficient users to manipulate settings that vary according to specific requirements. Consequently, most users rely on well-implemented slicer programs to facilitate object printing.

**Figure 2:**
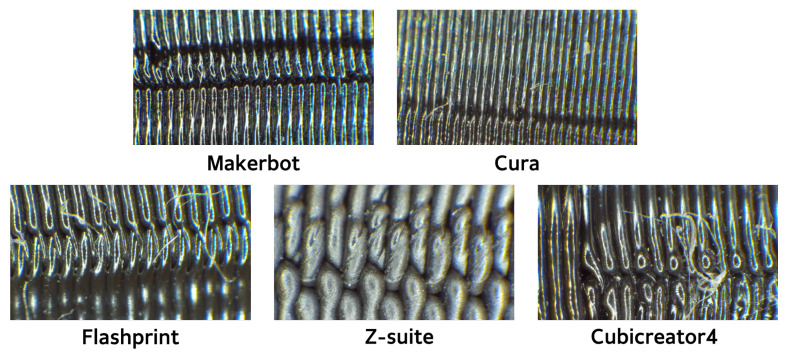
Distinctive output features of each slicer program using Z-seam.

Slicer programs slice models, thus dividing them into individual layers, and handle print settings in the software environment, such as output temperature, printing speed, layer thickness, shell count, and application of supports [23]. When aiming to print objects with high resolution, the layer thickness and shell count play a crucial role in achieving the desired consequence. A thinner layer results in less defined surface boundaries and smoother prints when dealing with curved surfaces. Conversely, an increase in the layer thickness results in rough surfaces. A lower layer thickness allows the precise representation of details but requires extensive support structures to handle significant overhangs. The residual marks remaining after the support removal can become indicators of the individuality and distinctive features of 3D printing. Moreover, 3D printers offer the flexibility to apply various slicer programs, each supporting different fine feature patterns, such as initial configuration, infill patterns, shell count, printing speed, starting positions, and support structure. The unique characteristic patterns inherent in each slicer program are manifested as “slicer fingerprints” on 3D objects, providing identifiable features. In particular, the Z-seam pattern that emerges when the nozzle transitions to the next layer is significantly influenced by the slicer program [24]. The Z-seam refers to the marks or imperfections that occur on the printed object when the stepper motor stops and the print head attempts to lift after processing the previous layer. These marks are caused by pressure, resulting in filament residue adhering to the printed object. Slicer programs modify the travel paths to effectively manage this inevitable residue, thus forming distinct patterns. Figure 2 shows the differences in the Z-seam characteristics between the slicer programs used in the experiments. All figures in this paper were created in high resolution, with an image resolution set at DPI 300. Each slicer program exhibited unique periodic patterns, as the G-code was designed differently to conceal the Z-seam.

**Figure 3:**
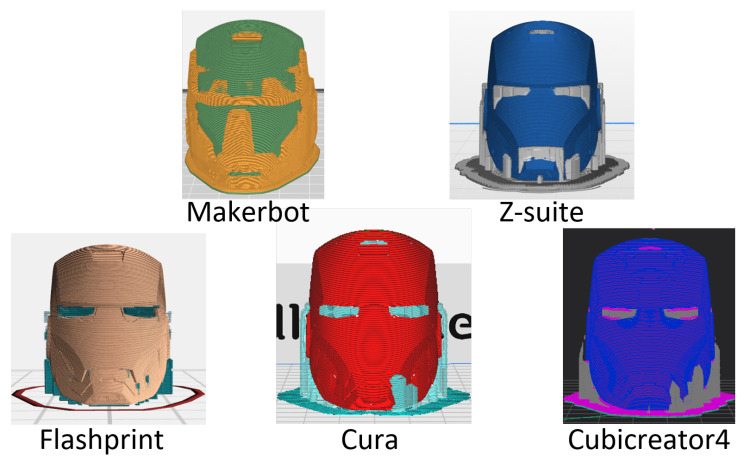
This figure shows that each slicing program exhibits different structures of supports or infill patterns.

Furthermore, the diversity in the shapes of the support structure reflects the uniqueness of each slicer program [32]. Structures such as overhangs or bridges may appear when complex or irregular models are used. Although the specific angles may vary depending on the material when the angle with respect to the vertical direction exceeds 45${}^{\circ }$, the weight of the overhang exceeds the rigidity of the material, leading to the collapse of the overhang. When printing 3D objects, the absence of support structures can result in low-quality results or even printing failures. Support structures are considered essential elements of the 3D printing workflow to address these issues. Figure 3 illustrates that support structures of various slicer programs differ in shape, resulting in scratch patterns that serve as “slicer fingerprints” when removing the supports from the printed 3D objects. Moreover, although not specifically addressed in this study, fill patterns, which vary among different slicer programs, serve as distinctive features for each program. Kubo et al. [17] demonstrated the successful identification of 3D printed objects based on the differences in fill patterns. In addition, fill patterns are utilized as artistic patterns and can be used as evidence to distinguish genuine items from counterfeits [33]. Table 1 summarizes the information regarding the slicer programs used in this experiment. While most slicer programs allow setting the layer thickness as continuous data, the Z-Suite used for M200, M300, and M300+ 3D printers specifies the layer thickness as a discrete variable. In addition, the available ranges of the configurable settings differ for each slicer program. This indicates the need for a detailed algorithm with a different approach than the classification methodology used for layer thickness identification.

#### 2.2.2. Hardware Domain

Upon receiving the G-code instructions, a 3D printer utilizes the hardware components for processes such as extrusion, material deposition, cooling, and solidification to print the 3D object. However, operational methods and component configurations can vary among various types of hardware serving the same purpose. Before initiating the printing process, all 3D printers must undergo a bed-leveling process to ensure even extrusion of the material across the entire printing bed surface. Although manual bed leveling is common in budget 3D printers, many 3D printers support automatic bed-leveling sensors developed by their respective manufacturers. Different material extrusion methods can leave pattern artifacts on the surface of a printed object. Direct-drive extrusion, in which the motor mount and hot end are directly connected, results in slower printing speed but higher quality of output. In contrast, the Bowden extrusion, where the motor mount and hot end are separated, allows for faster printing speed but may result in a lower quality of output. Furthermore, the diversity of the build plates, which significantly influences the material adhesion, results in different surface textures and characteristic patterns of printed objects.

A filament is an integral material for 3D printing. A wide variety of filaments is available for use in 3D printers. Despite printed objects having the same shape, each filament possesses unique properties, resulting in subtle differences in the processing method [34]. The printing temperature is the most critical parameter to consider, and the optimal temperatures and characteristics of the filaments are listed in Table 2. An improper configuration of the printing environment for ABS filaments can lead to significant thermal shrinkage and warping, which can adversely affect the thickness estimation of the layer. Additionally, in the case of the Method X printer, which utilizes dual extruders, the PVA filament used for support generation is water-soluble. This property allows the dissolution of the support in water without the need for physical force, resulting in minimal damage to the layer thickness and surface quality during support removal.

**Figure 4:**
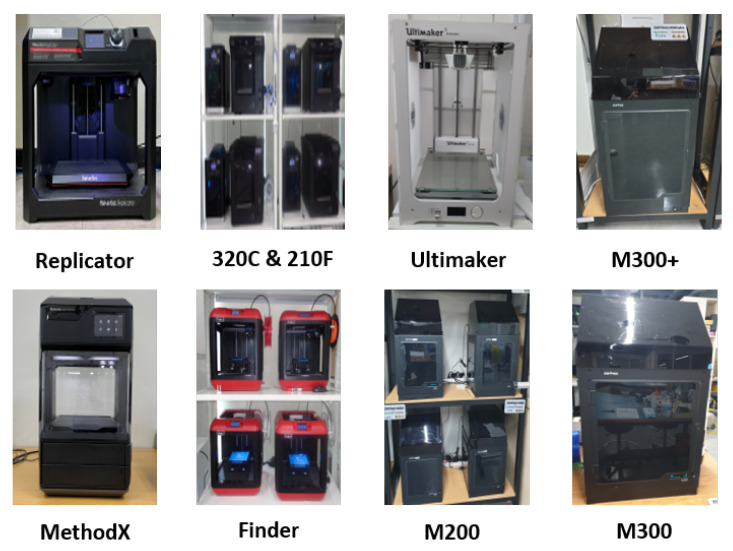
3D printers used for the SI3DP++ dataset output.

The 3D printer models, which are amalgamations of hardware components, are organized using their own intelligent sensors, depending on the manufacturing company and brand. Each printer model exhibits a different range of printing speeds, manifested as a periodic feature. The presence of a constant-temperature chamber within the 3D printer and variations in patents and applied technologies across different models give rise to different pattern noises that are consistent for a given model. The pattern noise is embedded in the surfaces of the printed 3D objects, thus providing evidence for identifying the source. Figure 4 shows the different types of 3D printers used in the layer thickness estimation task.

#### 2.2.3. Other Factors

Heretofore, we discussed pattern noise resulting from various features and components in the software and hardware domains. However, even objects printed in the same environment do not exactly match owing to variations in the conditions and specific details of the 3D printer, resulting in the occurrence of random noise. To achieve the high performance of sensor algorithms, it is essential to effectively capture and analyze these random noises, as they serve as crucial evidence in identification research [35].

The condition of the 3D printer varies with each iteration during the extrusion process, resulting in the random occurrence of tiny surface noises in the form of wave-like patterns. Three-dimensional printers prioritize maintaining an optimal printing temperature; however, variations in humidity and temperature owing to seasonal and weather changes make it difficult to achieve consistent output results. In addition, the presence of adhesive residues or remnants from previous prints can disrupt the pattern consistency. As observed by Aronson et al. [36], scratches or marks on a 3D printer are traces for source identification. These randomly occurring scratches can serve as fingerprints of the 3D printer. When conducting research on estimating the layer thickness through the surface imaging of printed objects, the output patterns play a significant role in determining the boundaries of each layer visible in the images. Ultimately, the consistent yet irregular patterns arising from various characteristics of the 3D printing process, such as specific components of the printer, slicer programs, and materials used, enable source identification in 3D printing. This underscores the need for more precise algorithms in 3D printing, reflecting the importance of capturing and analyzing these patterns.

## 3. Methods

### 3.1. Collecting Dataset

**Figure 5:**
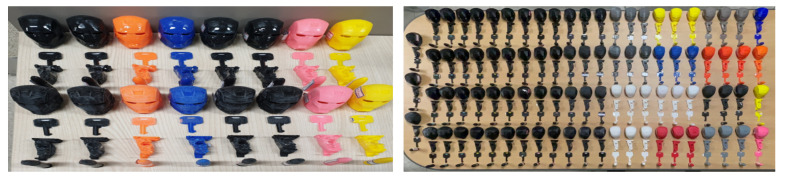
Samples of fabricated 3D objects for SI3DP++ dataset.

**Figure 6:**
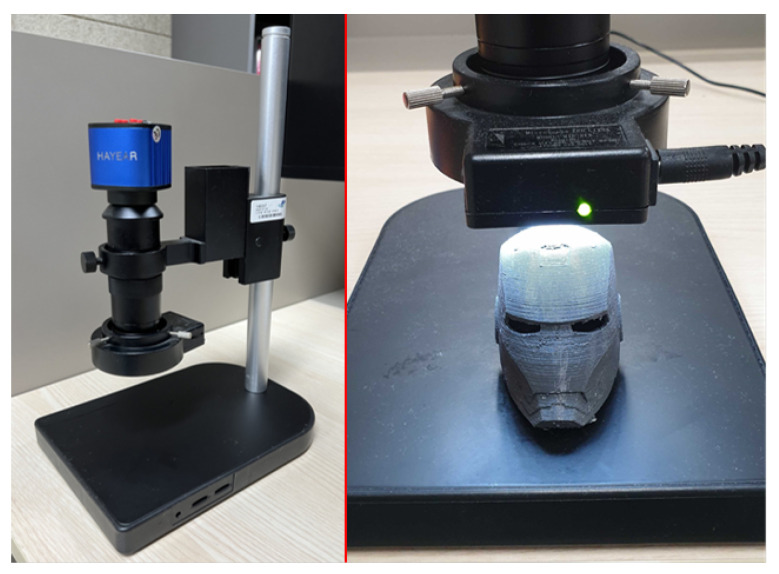
Optical microscope used for imaging the SI3DP++ dataset.

**Figure 7:**
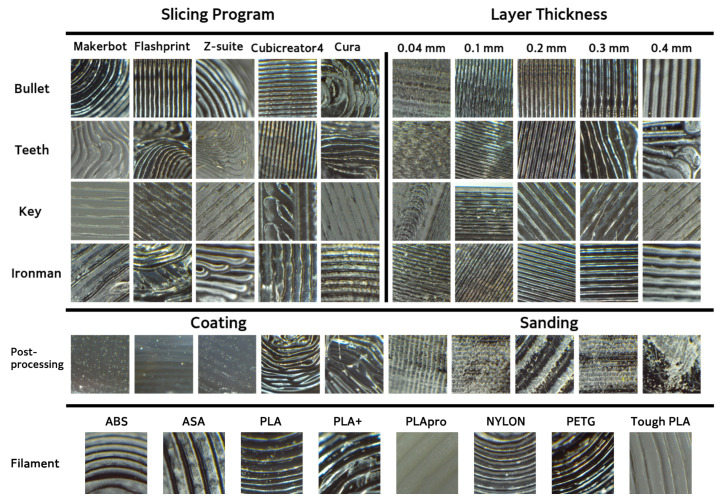
Samples from the SI3DP++ dataset.

**Figure 8:**
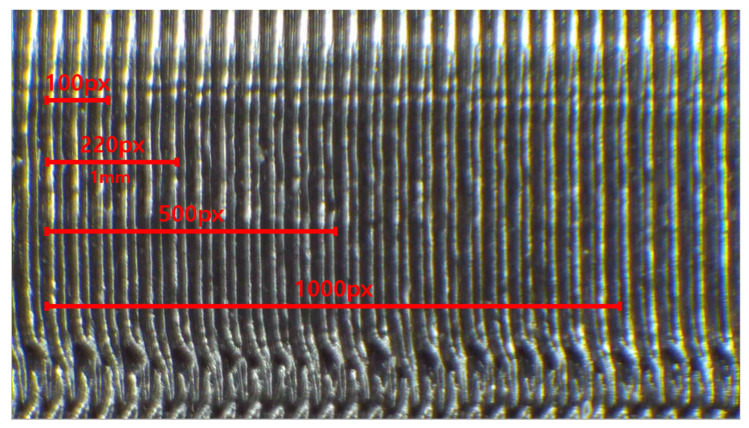
This figure visually depicts the mm-per-pixel ratio. The sample image is of a bullet object printed with a 210F printer, with a layer thickness set to 0.2 δ (mm). In the image resolution, one can see red lines indicating pixel lengths of 100, 500, and 1000, as well as a red line corresponding to 220 pixels, representing 1 δ (mm) which is equivalent to five 0.2 δ (mm) layers.

We used the SI3DP++ dataset [16] in our experiments. The SI3DP++ dataset is an open benchmark dataset specifically designed for 3D printing forensics. This dataset comprises close-up and full-shot image data captured using microscopy and digital cameras, showing objects printed under various environmental settings. To align with crime scenarios, such as copyright infringement, theft, forensic investigations, and homicides, 3D objects, such as Iron Man, a key, tooth, and bullet designs were selected as the output models. A total of 464 3D printed objects were produced (see Figure 5), with each object encompassing a minimum of 50 microscopy images. The microscope used for shooting was the HAYEAR HY5200 camera with an optical magnifier. The close-up images of SI3DP++ were captured using 50× optical zoom, representing the complex surface texture of 3D objects, and were saved as JPEG images with a resolution of 1280 × 720 pixels (see Figure 6). Detailed labeling, including printer type, filament type, layer thickness, shell count, device used, scanning and reprinting status, post-processing, and slicer program, was provided for each object based on the specific source identification tasks. The term “device” in the dataset refers to products with the same model but different serial numbers (e.g., 210F-1, 210F-2, 210F-3, 210F-4). Among these labels, this study focused on five types of layer thicknesses and five types of slicer programs for analysis. The values for printer, filament, and device played a crucial role as subtasks in the multi-task approach used for slicer program identification. For convenience, we utilized 32,080 close-up images from the SI3DP++ dataset, denoted as *I*. Sample photographs of the SI3DP++ dataset are shown in Figure 7. Moreover, Figure 8 visually depicts the relationship between the resolution of the images and their corresponding distances in the real world. To measure the exact length in pixels, we utilized the measurement function in HAYEAR software. The HAYEAR software used here allows for image capture, measurement, and editing, and is a program from the same company as the microscope utilized during the collection of the SI3DP++ dataset. As illustrated, the close-up images represent 1 mm for every 220 pixels, leading to an approximate mm-per-pixel ratio of 0.004545 for the SI3DP++ dataset.

Additionally, the SI3DP++ dataset includes a post-processing dataset. The post-processing consists of a sanding process that uses sandpaper to apply rough scratches to the object’s surface, and a coating process that utilizes Smooton’s XTC-3D epoxy coating solution to alter the surface texture of the object. The printer models used for post-processing are Cubicon’s 210F and 320C. We decided to utilize the post-processing dataset to diversify the training data.

### 3.2. Source Attribution Analysis

#### 3.2.1. Slicer Program Identification

Slicer programs are primarily designed to enhance user convenience during 3D printing. However, because of their varying marketing objectives, they exhibit visible differences in interfaces, print settings, compatibility, and other aspects. Conversely, differences in inherent attributes such as infill patterns, support structure shapes, and boundary artifacts manifest as hidden characteristics of the 3D output. Ultimately, each slicer software is programmed in different ways to enhance the quality and strength of the output [23,24]. However, there is a lack of research on identification of slicer programs. We examined the classification results of five types of slicer program: Makerbot, Cubicreator4, Flashprint, Z-Suite, and Cura. For reference, the printer types used for each slicer program were as follows: Makerbot data from Method X and Replicator, Cubicreator4 data from 210F and 320C, Flashprint data from Finder, Z-Suite data from M200, M300, and M300+, and Cura data from Ultimaker.

#### 3.2.2. Layer Thickness Estimation

A primary reason for adjusting the layer thickness setting is to enhance the resolution of the output while minimizing the printing time, with the aim of achieving a balanced and high-quality result. The periodic patterns that manifest on the outer surface of a 3D object are closely related to the layer thickness and are more visibly discernible than other parameters. Consequently, previous studies have focused on layer thickness identification using classification methods. However, as shown in Table 1, most products have continuous variable parameter settings for the layer thickness, making it impossible to achieve detailed source identification of the output values using traditional methods. Therefore, we researched the layer thickness estimation based on a regression model. For our study, we selected the following five parameters from the SI3DP++ dataset: Layer1 (0.06 mm), Layer2 (0.1 mm), Layer3 (0.2 mm), Layer4 (0.3 mm), Layer5 (0.4 mm). Here, δ (mm) denotes the layer thickness.

### 3.3. Proposed Methods

In our experiments, we employed the ViT model, which has attracted significant attention in the field of computer vision. Unlike convolutional neural networks (CNNs), ViT utilizes attention mechanisms to enable global interactions within an image, making it suitable for capturing the relationships between features across a wide range. We utilized a pretrained CoaT-Lite Tiny transformer model [28] as our base network. We defined the layer thickness and slicer program tasks as T and S, respectively. For each task, we constructed an estimation model by employing different loss functions and classifiers. The objective function is defined as follows:

(1) $$\begin{array}{cc} L_{T}= & L_{MSE}\left(R_{{}_{T}}\left(E\left(I\right)\right)\right),\\ L_{S}= & \beta \;\cdot \;L_{CE}\left(C_{{}_{S}}\left(E\left(I\right)\right)\right)\\ & +\left(1-\beta \right)\;\cdot \;L_{CE}\left(C_{\hat{\theta }}\left(\left[E\left(I\right)\right]\right)\right), \end{array}$$

where $L_{T}$ represents the regression loss for the layer thickness estimation and $L_{S}$ represents the classification loss for slicer program identification. $L_{MSE}$ and $L_{CE}$ denote the mean squared error (*MSE*) loss and cross-entropy losses, respectively. *E* denotes the encoder for feature extraction from the images, and *R* and *C* denote the regressor and classifier, respectively. $\hat{\theta }$ refers to the subtasks for multitask learning that represent the information on the printer, filament, and device in the SI3DP++ dataset. In our multitask learning approach, we prioritized the main task by setting the value of β to 0.8. In addition, we employed a data fusion technique to integrate the information from multiple angles for a single 3D object. This process helped us create a unified representation for each 3D object by averaging the logit values from multiple image angles. As for the averaging of logit values, we utilized the unique IDs assigned to each 3D object in the SI3DP++ dataset. Each object was given a distinct identifier, and we averaged the logit values based on these unique IDs. This averaging procedure ensures that the model’s predictions are consistent and comparable across different objects, regardless of their individual characteristics or appearances. An Adam optimizer with a learning rate of 1 × 10${}^{-5}$ was used, and the model was trained for 30 epochs. To address the limitations of the dataset, we employed a 4-fold cross-validation technique for data augmentation and model evaluation. We set the input image size to 224 in order to apply the pre-trained weights of the ViT model.

### 3.4. Evaluation Metrics

Classification and regression problems generally involve different evaluation methods. Classification aims to determine how accurately a model classifies data. Therefore, performance metrics such as accuracy, precision, recall, F1-score, and confusion matrix are used. In contrast, regression focuses on how accurately a model predicts the target values for the input data by using metrics such as the root mean squared error (RMSE) and R2-score.

In our study, we employed the regression-to-classification metrics to evaluate the performance of the regression prediction model in comparison with that of the classification model. The classification regression metric involves mapping the final output indices from the classification model to the actual layer thickness labels. This transformation allowed us comparison of the output values of the classification model with the ground truth labels and calculation of the RMSE score. The related equations are given below.

(2) $$\begin{array}{cc} Z_{l}\;= & C_{T}\left(E\left(I\right)\right),\\ Z_{p}= & argmax(softmax\left(Z_{l}\right)),\\ Z\;\;\;= & f(Z_{p}),\\ RMSE= & \sqrt{\frac{1}{n}\sum_{i=1}^{n}{\left(y_{i}-Z_{i}\right)}^{2}}, \end{array}$$

where $C_{T}$ represents the classifier trained for layer thickness identification, and *f* is the function that maps the final predicted index obtained through the argmax function to the corresponding actual label value. *y* denotes the values ($y_{i}=0.06,0.1,0.2,0.3,0.4$). $Z_{l}$ and $Z_{p}$ denote the feature vectors of the classification model corresponding to task *T* and the final index value after passing through the softmax function, respectively. The RMSE score was calculated by comparing the final *Z* values with the actual labels and computing the error. To demonstrate the efficiency of the regression model for task *T*, the 0.2 δ(mm) dataset was not utilized during the training and validation phases. Furthermore, experiments related to layer thickness estimation were evaluated using a dataset comprising all layers, including the 0.2 δ(mm) layer. This allowed us assessment of the overall performance across all layers, rather than specific layers when comparing the performance of classification and regression models. Because we used a classification model, we utilized precision as the evaluation metric for the slicer program task.

## 4. Results

We present the experimental results for layer thickness estimation and slicer program identification in Table 3 and Table 4, respectively. The regression approach shows superior performance in layer thickness estimation. We also demonstrate its ability to identify the slicer programs. Additionally, Table 5 presents a comparison of the experimental results with those of previous studies. A detailed discussion of the results is presented in the following section.

### 4.1. Layer Thickness Estimation

To demonstrate the effectiveness of the regression models for layer thickness estimation, we conducted experiments using both the classification and regression models. In addition, we compared the performance of the CNN-based- and transformer-based models in regression tasks. The results, summarized in Table 3, show that the error of the regression approach was significantly lower by approximately 0.025 when compared with that of the classification approach, indicating a notable performance difference. Moreover, the error of the transformer-based CoaT-Lite Tiny model [28] was approximately 0.004 lower than that of the CNN-based EfficientNet-B3 model [37]. Unlike CNN-based models, which primarily focus on learning local features, transformer models leverage the self-attention mechanism and hierarchical representation to capture the overall image features. For the regression model, we could observe that there was no a significant difference in the RMSE when comparing the Ironman and Teeth items to the mean value. The Bullet, which had a less complex shape, showed reduced errors compared to the mean value. However, for pieces like the Key, which occupies a substantial portion of the bottom surface relative to the overall area, we could observe larger errors. This was due to excluding significant support removal artifacts on the bottom surface when collecting the SI3DP++ dataset. Nevertheless, the regression model showed to be effective in estimating layer thickness for all cases. This enabled them to achieve a more accurate and generalized estimation of the thickness of the final layers.

Additionally, we conducted comparative experiments for the classification model and the regression model using different thicknesses as test cases. Table 6 illustrates the result of training the model with data excluding the layer thickness corresponding to each target and then evaluating its performance using the data from the excluded layers as the test set. For most layer thicknesses, excluding 0.2 mm and 0.3 mm, the classification model performed well. This showed that the regression model, being data-driven, struggled with extreme thickness prediction task. However, for thicknesses like 0.2 mm and 0.3 mm, which fall within the middle of the label range, the RMSE scores demonstrated the superior performance of the regression model. This indicated that the regression model has an advantage in predicting thicknesses within the entire label range, even for data it does not encounter during training. Additionally, given that 3D printers can produce objects with continuous variables, this advantage extends to generalization in real-world settings.

### 4.2. Slicer Program Identification

**Figure 9:**
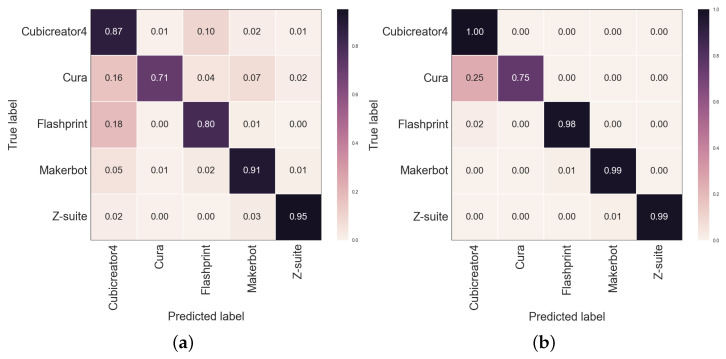
Confusion matrices for slicer program identification: (**a**) basic model and (**b**) basic model + printer + data fusion.

Table 4 summarizes the performance results for slicer program identification. The precision score for the basic model without a data handling technique was 0.88. In addition, the multitasking performance of the printer and filament showed an improvement of approximately 0.01. This improvement can be attributed to the relevance of the printer and filament information in designing support structures and generating Z-seams in the slicer program, which allows the model the learning of shared features and achievement of better performance. However, information regarding the device primarily focuses on its specific characteristics, thus limiting the performance of identifying slicer programs. Finally, the model utilizing the data fusion technique achieved a precision score of 0.987. This indicates that capturing comprehensive feature information from images of the same 3D object at different angles enables a better overall performance. To examine the identification results of each slicer program, the confusion matrices for the basic and final models are presented in Figure 9. It can be observed that the final model performed better than the basic model in classifying Flashprint and Cura slicers.

### 4.3. Comparison Result

We compared our performance results with those of existing studies. Li et al. [15] proposed a GLCM-SVM-based classification model that captures the interpixel correlation within images. However, their model exhibited an error of 0.099354 in terms of the RMSE score. Shim et al. [21] conducted their research using a CNN-based classification model by employing a multimodal approach for close-up and full-shot images. In addition, Shim et al. [16] proposed a two-stream texture encoder, referred to as CFTNet, combined with fast Fourier transform and positional encoding of the transformer encoder. However, their proposed model yielded slightly higher results of approximately 0.025 when compared with our proposed model. Another study by Hou et al. [38] focused on the layer thickness estimation; however, they utilized scanned three-dimensional digital data of the output objects, which differs from our approach. In conclusion, our proposed regression approach and transformer-based model outperformed existing studies in layer thickness estimation.

## 5. Discussion and Conclusions

This study researched layer thickness estimation and slicer program identification by analyzing the characterization factors that arise in the hardware and software domains of the 3D printing process. Previous studies [16,21] on layer thickness estimation have approached this problem through classification based on labels. However, because each layer thickness parameter can be defined as a continuous variable during the parameter setting, we proposed an estimation model based on regression methods. Through comparative experiments of various models and methodologies, we observed that the task of layer thickness estimation was more effectively addressed by applying regression methods based on the ViT model. Furthermore, by comparing our research on layer thickness estimation with previous 3D printing forensic studies [15,16,21], we were able to quantitatively assess that our new methodology is more effective. Our proposed model is capable of handling a wider range of data than those in previous research, and demonstrates higher performance than the classification models.

In addition, we revealed a novel research direction by presenting a method for identifying slicer programs. We analyzed the differences in the output characteristics of 3D slicers and conducted research to identify 3D slicers, discovering that classification is possible. This has implications for the investigation of potential risks associated with 3D printers and criminal cases involving 3D printing. It is important to note that 3D printed objects used for illegal purposes, such as 3D guns, require a high level of precision and accuracy to withstand live ammunition. With that in mind, the identification of the layer thickness and slicer programs is crucial for understanding the potential risks and addressing issues related to 3D printing. Accurate parameter estimation also assists in distinguishing between counterfeit and genuine products, and enhances product identity and authenticity.

The limitations of our study are as follows. In this study, we utilized the SI3DP++ dataset, which is an open dataset focused on 3D printing identification. However, the SI3DP++ dataset had only five classes, and it did not cover a broader and more detailed range of layer thickness, which was needed for our precise layer thickness estimation task. Furthermore, relying solely on publicly available datasets for research on source printer identification is limited, particularly in tasks involving the identification of different slicer programs used by the same printer. On the other hand, applying our research methodology to alternative 3D printing methods like SLA, SLS, and DLP presents challenges due to their distinct principles, materials, and operational characteristics [39,40,41]. SLA’s thin layer thickness [42] and SLS’s rough surfaces [41] make adapting our approach difficult. Each technique’s unique attributes pose obstacles in translating our methodology to different methods. Therefore, comprehensive research in diverse environments and specific conditions is needed. We suggest the collection of more detailed datasets for the advancement of 3D printing forensics, leaving open possibilities for future research.

## Figures and Tables

**Table 1:** Summary of slicer programs used in the study.

Slicer Program	3D Printer	Layer Thickness Range (mm)	Printing Speed (mm/s)
Makerbot	Method X	0.2–1.2	10–500
	Replicator		
Cubicreator4	210F	Continuous variable	5–500
	320C		
Flashprint	Finder	0.05–0.4	10– 200
Z-suite	M200		50–200
	M300	0.09, 0.14, 0.19,	
	M300+	0.29, 0.39	
Cura	Ultimaker	Continuous variable	Continuous variable

**Table 2:** Filament types used in the SI3DP++ dataset.

Name	Temp.	Summary
PLA	210 °C	Polylactic Acid. Most widely used filament. Biodegradable material, non-toxic, no flexibility, weakens under humidity
PLA+	210 °C	Similar composition as that of PLA but has 3 times higher stiffness
ABS	245 °C	Acrylonitrile butadiene styrene. Very sturdy, financial, emits harmful gas during printing
Tough PLA	210 °C	Add properties of ABS with elasticity and stiffness to PLA. High toughness, greater durability
Nylon	250 °C	Flexible, has greater elasticity and gloss than PLA
PETG	230–250 °C	Polyethylene Terephthalate Glycol. Smooth surface finish, high transparency, water/chemical resistance
ASA	245 °C	Acrylonitrile Styrene Acrylate. Material that combines the advantages of ABS with UV and moisture resistance
PLA pro	230 °C	Industrial PLA with high speed performance, good mechanical properties, and adaptability to high-heat environments

**Table 3:** Performance results for the layer thickness estimation experiment.

Approach	Method	Bullet	Iron Man	Key	Teeth	Mean	Standard Deviation
Classification	CNN	0.0765	0.0787	0.0804	0.0759	0.0779	0.0021
Classification	Transformer	0.0759	0.0793	0.0798	0.0814	0.0791	0.0023
Regression	CNN	0.0451	0.0538	0.0702	0.0535	0.0564	0.0105
Regression	Transformer	0.0374	0.0523	0.0701	0.0461	0.0529	0.0138

CNN: EfficientNet-B3 [37], Transformer: CoaT-Lite Tiny [28], Unit: δ (mm).

**Table 4:** Slicer program identification performance.

Method	Subtask Type	Fusion	Precision
CoaT-Lite Tiny [28]	✗	✗	0.880
	Filament	✗	0.888
	Device	✗	0.880
	Printer	✗	0.889
CoaT-Lite Tiny [28]	Printer	✓	0.987

**Table 5:** Comparison of experimental performance for layer thickness estimation.

Model	Approach	Description	Rmse Score
Li et al. [15]	Classification	GLCM-SVM	0.0994
Shim et al. [21]	Classification	CNN-based	0.0791
Shim et al. [16]	Classification	CFTNet	0.0773
Ours	Regression	Transformer-based	0.0529

**Table 6:** Performance results of the comparative experiments for different layer thickness estimation.

Approach	Method	0.06 δ (mm)	0.1 δ (mm)	0.2 δ (mm)	0.3 δ (mm)	0.4 δ (mm)
Classification	CoaT-Lite Tiny [28]	0.0781	0.1034	0.1063	0.1122	0.1425
Regression	CoaT-Lite Tiny [28]	0.0961	0.1251	0.0615	0.0963	0.1676

## Data Availability

Not applicable.
